# Cost analysis of implementing HIV drug resistance testing in Kenya: a case study of a service delivery site at a tertiary level hospital in Kenya

**DOI:** 10.12688/f1000research.23379.1

**Published:** 2020-07-29

**Authors:** Rachael W. Gachogo, Daniel N. Mwai, Frank G. Onyambu

**Affiliations:** 1 Molecular and Infectious Diseases Research Laboratory, University of Nairobi, Nairobi, Kenya; 2 School of Economics, University of Nairobi, Nairobi, Kenya; 3 School of Health Sciences, Meru University of Science and Technology, Meru, Kenya

**Keywords:** HIV, HIV drug resistance testing, implementation science, cost analysis, health systems strengthening

## Abstract

**Background:** HIV drug resistance (HIVDR) threatens progress achieved in response to the HIV epidemic. Understanding the costs of implementing HIVDR testing programs for patient management and surveillance in resource-limited settings is critical in optimizing resource allocation. Here, we estimate the unit cost of HIVDR testing and identify major cost drivers while documenting challenges and lessons learnt in implementation of HIVDR testing at a tertiary level hospital in Kenya.

**Methods: **We employed a mixed costing approach to estimate the costs associated with performing a HIVDR test from the provider’s perspective. Data collection involved a time and motion study of laboratory procedures and interviewing laboratory personnel and the management personnel. Cost analysis was based on estimated 1000 HIVDR tests per year. Data entry and analysis were done using Microsoft Excel and costs converted to US dollars (2019).

**Results: **The estimated unit cost for a HIVDR test was $271.78 per test. The main cost drivers included capital ($102.42, 37.68%) and reagents (101.50, 37.35%). Other costs included: personnel ($46.81, 17.22%), utilities ($14.69, 5.41%), equipment maintenance costs ($2.37, 0.87%) and quality assurance program ($4, 1.47%). Costs in relation to specific laboratory processes were as follows: sample collection ($2.41, 0.89%), RNA extraction ($22.79, 8.38%), amplification ($56.14, 20.66%), gel electrophoresis ($10.34, 3.80%), sequencing ($160.94, 59.22%), and sequence analysis ($19.16, 7.05%). A user-initiated modification of halving reagent volumes for some laboratory processes (amplification and sequencing) reduced the unit cost for a HIVDR test to $233.81 (13.97%) reduction.

**Conclusions: **Capital expenditure and reagents remain the most expensive components of HIVDR testing. This cost is bound to change as the sequencing platform is utilized towards maximum capacity or leveraged for use with other tests. Cost saving in offering HIVDR testing services is also possible through reagent volume reduction without compromising on the quality of test results.

## Background

Unprecedented increased access to antiretroviral therapy (ART) is one of the greatest milestones in the fight against the HIV epidemic, resulting in reduced mortality from AIDs-related causes and a global decline in HIV incidence ( UNAIDS, 2019). However, this success is threatened by emergence of HIV drug resistance (HIVDR). The World Health Organization (WHO) reports greater than 10% pretreatment drug resistance to non-nucleoside reverse transcriptase inhibitors (NNRTIs) among adult patients starting on a first-line ART regimen. This rate is higher in children below 18 months, with over half of newly diagnosed infants harboring resistance to NNRTIs. The prevalence of acquired HIVDR among patients on ART ranges from 3% to 29% ( WHO, 2019). Moreover, recent studies in Kenya have shown an upward trend in both transmitted and acquired HIVDR ( Hassan *et al.*, 2019; Kantor *et al.*, 2018; Milne *et al.*, 2019).

ART is delivered through the public health approach in most low- and middle-income countries, where standardized drug regimens are administered with simplified laboratory monitoring using tests such as HIV viral load and CD4 count assays ( Lessells *et al.*, 2013; De Luca *et al.*, 2013; Phillips *et al.*, 2018). Access to HIVDR testing is limited for patients in resource-limited settings such as Kenya due to high costs involved and inadequate laboratory capacity ( Kennedy *et al.*, 2016; Nkengasong *et al.*, 2018; Petti *et al.*, 2006). On the other hand, HIVDR testing is offered routinely in resource-rich setting to inform clinical management of people living with HIV ( Dunn *et al.*, 2011; Günthard *et al.*, 2019). However, considerable effort has been made to monitor population level of HIVDR in low- and middle-income countries by implementing HIVDR surveys according to WHO guidelines. These surveys have been crucial in informing national ART guidelines; for example, data on the high prevalence of pretreatment drug resistance to NNRTIs has been critical in the transitioning from an NNRTI-based first-line regimen to a regimen that consists of dolutegravir (DTG) in sub-Saharan countries ( WHO, 2019).

Funding for HIV programming in Sub-Saharan countries is provided by multilateral development partners including the US President’s Emergency Plan for AIDs Relief (PEPFAR), the Global Fund and the World Bank, among others ( Ministry of Health, 2015; Mwai, 2017; Olakunde *et al.*, 2019; Schneider *et al.*, 2016). These multilateral partners have acknowledged the integral role played by quality medical laboratory services within health systems, precipitating mobilization of significant amounts of funding earmarked for laboratory systems strengthening in resource-limited settings ( Abimiku, 2009; Hamel *et al.*, 2015; Nkengasong *et al.*, 2018). This support has led to great improvement in HIV diagnostic and monitoring services, inter-laboratory networks, systems, governance, and institutions, evident by the increased number of laboratories that have the capacity to perform molecular diagnostics for HIV viral load and early infant diagnosis (EID), as well as HIV genotyping. Furthermore, these partners have been at the forefront in supporting development of national laboratory policy and strategic plans, and improving quality in these laboratories ( Nkengasong *et al.*, 2018; Hamel *et al.*, 2015).

In Kenya, there are 10 laboratories across the country that support HIV viral load and EID testing through a PEPFAR and Global Fund funded specimen referral network, and only four of these have capacity to perform HIVDR testing. However, there is a paucity of costing data from these laboratories and no detailed cost analysis has been done ( Inzaule *et al.*, 2013). Some of the essential costs when performing a costing analysis include equipment costs, personnel costs, and utilities costs. Previous studies have included reagents cost and consumables cost in their estimations, excluding major cost categories ( Acharya *et al.*, 2014; Inzaule *et al.*, 2013; Novitsky *et al.*, 2015; Zhou *et al.*, 2011). A detailed cost analysis provides a deeper understanding of the costs of HIVDR to the health systems. Moreover, cost information is important in development of business plans, projections, planning, budgeting, pricing and resources allocation. Finally, it gives a good guide on the affordability of HIVDR testing inclusion in the standard package of HIV testing in Kenya to alleviate the rising number of cases of HIVDR.

In this study we estimate the unit cost of HIVDR testing, identify the cost drivers for the HIVDR test, explore opportunities for cost saving and document challenges and lessons learnt in implementation of HIVDR testing.

## Methods

### Ethical statement

Ethical approval was obtained from University of Nairobi/Kenyatta National Ethics Review Committee (KNH-UON-ERC- P562/01/2019). Verbal informed consent was obtained from all participants for the interviews and the ethics review board waived the need for a signed consent form. the study followed the guidelines for verbal consent, including explaining to the study participants the pertinent issues about the study including the purpose, benefits, risks and procedures. The participants were also given enough time to decide whether to participate or not as well as an opportunity to ask questions.

### Study site

This costing study was conducted at the Molecular and Infectious Diseases Research Laboratory (MIDRL) located within the Kenyatta National Hospital and University of Nairobi School of Medicine. The MIDRL supports the implementation of high quality, sustainable and comprehensive HIV prevention, care and treatment in Nairobi through provision of laboratory services including HIV viral load, HIV early infant diagnosis and HIVDR testing. Data collection was performed during the initial implementation stages, that is within the first year of commencing HIVDR testing.

### Costing methodology

The study utilized both micro and gross costing in quantification and valuation of the cost categories, which was done from the provider’s perspective.

### Data collection and analysis

Data were collected and compiled from 1 ^st^ January to 30 ^th^ June 2019. Costs were assessed for various processes in the HIVDR testing workflow including laboratory administration, sample collection and preparation, viral RNA extraction, nucleic acid amplification, gel electrophoresis, Sanger sequencing, data analysis and reporting. At the time of data collection, the laboratory used a commercial HIVDR assay supplied by Thermofisher (Cat. no. 12183018A, Waltham, USA). Cost data were collected on annualized depreciation for capital items including laboratory equipment and furniture, long term training and information technology equipment at a depreciation rate of 10%, reagents and consumables, personnel, utilities, laboratory and office space, quality assurance program, and maintenance costs. Cost details were obtained from quotations, invoices and delivery notes.

Data was collected by RG, who at the time of the study was working at MDRL as a clinical laboratory assistant and a master’s student (Health Economics and Policy) at the University of Nairobi. Data collection involved a time and motion study of the laboratory procedures for HIVDR testing and interviewing of laboratory and management personnel. The time and motion study was carried out in 12 sessions lasting between 1–3 hours based on the length of the laboratory procedure. A structured questionnaire depicting all the HIVDR testing steps and data collection tables were used to document quantity of reagents and consumables used as well as duration of each HIVDR process ( Gachogo *et al.*, 2020b). Two laboratory technical staff and one member of management were interviewed in three sessions (1 hour) each. Individuals with in-depth knowledge on HIVDR testing implementation were purposively selected to participate in the interviews. Interviews took place within their work environment. An interview guide was used to conduct the interview process and the data was recorded in form of field notes ( Gachogo *et al.*, 2020b). Technical staff were interviewed about HIVDR testing processes and experiences of implementing testing, while the member of management was interviewed about cost data, which was recorded in the data collection tables. None of the interviewees declined to participate. Administrative records such as invoices, requests for quotations and delivery notes obtained from the program archives were reviewed to obtain purchase costs. In addition, the laboratory personnel were asked to narrate their experience in setting up a HIVDR testing laboratory, with particular interest on problems encountered and solutions to these set-backs.

### Data analysis

We developed a Microsoft Excel version 2010 based model to aid in estimation of the unit cost and cost for the various categories and laboratory processes. All costs were converted to US dollars (13 ^th^ April 2019, $1 USD = 101.2 Kshs). The MIDRL projected to perform 1000 tests in 2019 based on the number of HIVDR tests performed in the first and second quarter of the 2019 financial year. Responses on challenges experienced during the implementation of HIVDR testing were manually scanned through by RG and FO for developing themes and coded accordingly.

### Sensitivity analysis

One-way sensitivity analysis for 20% variations to cost categories was performed to establish the level of uncertainty linked with costs variation of inputs to HIVDR test. This involved varying capital, personnel, reagents, maintenance, and quality assurance program costs by ±20% and evaluating how each of them influence the HIVDR unit cost relative to the estimated cost.

## Results

### HIVDR testing process

HIVDR testing is carried out in five major processes; namely, sample collection and preparation, nucleic acid extraction, nucleic acid amplification, sequencing and sequence analysis. Specimen collection and preparation involves collection of whole blood from the patient into blood collection tubes that contain ethylenediaminetetraacetic acid (EDTA) anticoagulant. Once the blood is collected into the blood collection tubes, the specimen is prepared for storage by spinning, pipetting and aliquoting into storage vials. The second step in HIV resistance testing is nucleic acid extraction from the plasma. In this step, HIV ribonucleic acid (RNA) is isolated from plasma. Once extracted and purified, the nucleic acid is converted to complementary deoxy-ribonucleic acid (cDNA) and amplified by polymerase chain reaction (PCR) and sequenced. Sequencing involves amplicon purification, cycle sequencing, amplicon purification, sequence detection and visualization. The last step in HIVDR testing is sequence analysis, which involves sequence data validation, sequence assembly, interpretation and quality analysis.

### HIVDR unit cost

**Figure 1.**
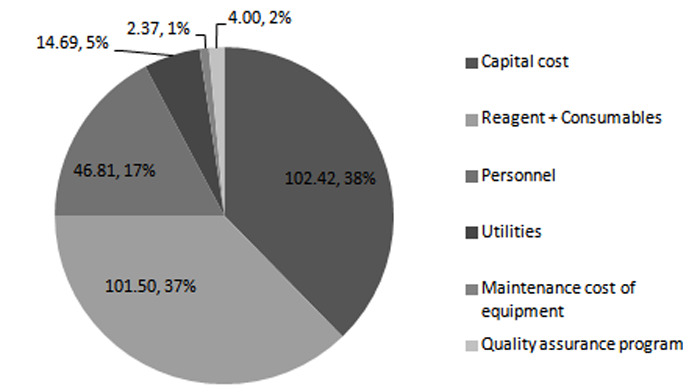
Distribution of cost of HIV drug resistance testing in capital, reagents + consumables, personnel, utilities, maintenance cost of equipment, and quality assurance program categories. Unit cost is $271.8.

Activity-based costing for HIVDR testing was performed at MIDR Laboratory by collecting cost data for each step in the drug resistance testing. The cost for performing HIVDR testing was US$ 271.78 per test, where capital costs took the biggest share at $102.42, followed by reagents and consumables at $101.5 ( Table 1 and Figure 1). Other costs included personnel ($46.81), utilities ($14.69), maintenance cost of equipment ($2.37) and quality assurance program ($4.00) ( Gachogo *et al.*, 2020a).

**Table 1.** Cost breakdown for each category. Costs in USD.

Item	Cost per test	%
Capital cost *	102.42	37.68
Reagents + consumables	101.48	37.35
Personnel	46.81	17.22
Utilities	14.69	5.41
Maintenance cost of equipment	2.37	0.87
Quality assurance program	4.00	1.47
**Total cost**	**271.78**	**100.00**

* The most costly component of HIV resistance testing.

### Cost per laboratory process

**Figure 2.**
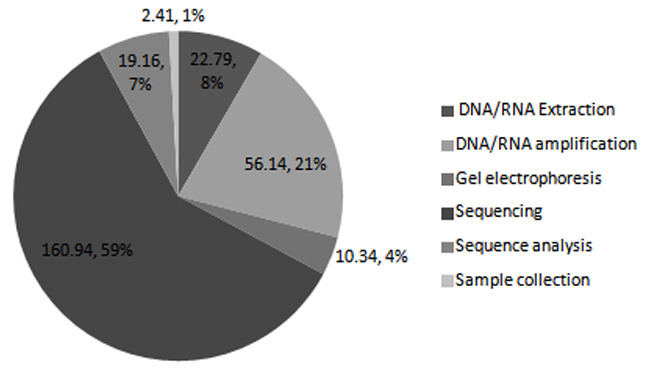
Distribution of costs of HIV drug resistance testing laboratory processes, including DNA/RNA extraction, DNA/RNA amplification, gel electrophoresis, sequencing, sequence analysis, and sample collection. Unit cost is $271.78.

The sequencing step had the largest cost of $160.94 per test, while DNA/RNA amplification had the second largest cost of $56.14. DNA/RNA extraction, gel electrophoresis, sequence analysis and sample collection had a cost of $22.79, $10.34, $19.16 and $2.41, respectively ( Table 2 and Figure 2).

**Table 2.** Cost breakdown by laboratory processes. Costs in USD.

Item	DNA/RNA extraction	DNA/RNA amplification	Gel electrophoresis	Sequencing *	Sequence analysis	Sample collection	Total cost
Capital cost	7.66	4.48	6.55	82.57	1.21	0.10	**102.56**
Reagents + consumables	5.42	37.30	1.47	55.63	0	1.67	**101.48**
Personnel	4.68	9.36	1.87	15.44	15.44	0.47	**47.27**
Utilities	4.39	3.96	0.20	5.20	0.41	0.11	**14.28**
Maintenance cost of equipment	0.24	0.24	0.09	0.78	0.78	0.02	**2.15**
Quality assurance program	0.40	0.80	0.16	1.32	1.32	0.04	**4.03**
**Total cost in** **USD**	**22.79**	**56.14**	**10.34**	**160.94**	**19.16**	**2.41**	**271.78**
**% Total cost**	**8.38**	**20.66**	**3.8**	**59.22**	**7.05**	**0.89**	**100**

* The most expensive step in HIV drug resistance testing.

### Cost of the modified HIVDR assay

**Figure 3.**
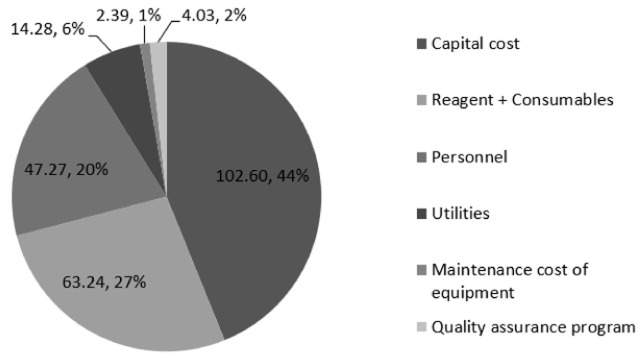
Cost distribution of the modified assay among HIV drug resistance processes including capital, reagents + consumables, personnel, utilities, maintenance cost of equipment and quality assurance program. Total unit cost is $233.81.

The laboratory validated a low-cost assay, whereby reagents volumes used during the amplification and sequencing steps were half the recommended volumes by the manufacturer. The test performance was in agreement with the original assay as previously reported ( Magomere *et al.*, 2019). The unit cost as a result of halving reagents volumes at the amplification and sequencing step was $233.81, a reduction from $271.78 of the original assay. There was a notable reduction for the amplification and sequencing costs to $38.38 and $140.70 from $56.14 and $160.94, respectively. There was no change in costs for the other steps in HIVDR testing ( Table 3 and Figure 3).

**Table 3.** Cost breakdown of the modified assay.

Item	DNA/RNA extraction	DNA/RNA amplification	Gel- electrophoresis	Sequencing	Sequence analysis	Sample collection	Total cost
Capital cost	7.66	4.48	6.55	82.57	1.24	0.1	102.60
Reagents + consumables	5.42	19.3	1.47	35.38	0	1.67	63.24
Personnel	4.68	9.36	1.87	15.34	15.44	0.47	47.27
Utilities	4.39	3.96	0.2	5.2	0.41	0.11	14.28
Maintenance cost of equipment	0.24	0.47	0.09	0.78	0.78	0.02	2.39
Quality assurance program	0.40	0.8	0.16	1.32	1.32	0.04	4.03
Total cost in USD	**22.79**	**38.38** ^1^	**10.34**	**140.70** ^2^	**19.16**	**2.41**	**233.81** ^3^
**% Total cost**	**9.74**	**16.41**	**4.42**	**60.18**	**8.21**	**1.03**	**100**

^1^ Halving reagent volumes at DNA/RNA amplification step reduces the cost of this step from $56.14 to $38.38.

^2^ Halving reagent volumes at sequencing step reduces the cost of this step from $160.94 to 140.70.

^3^ Halving reagent volumes at DNA/RNA amplification and sequencing steps reduce the HIV drug resistance test cost to $ 233.81 from $ 271.78.

### Cost of HIVDR test using US Food and Drug Administration (FDA)-approved assay

The cost for the HIVDR test using Viroseq HIV genotyping reagents and consumables (Abbott Molecular, Abbott Park, IL) was estimated at $379.46. This is one of the FDA-approved HIVDR tests available on the market and is used as an alternative to in-house reagents and consumables manufactured by Thermofisher. Reagents and consumables accounted for 55% ($209.18) of the unit cost of the HIVDR testing ( Table 4).

**Table 4.** Costs for HIV drug resistance test using US Food and Drug Administration approved reagents (Viroseq HIV genotyping). Costs in USD.

Item	Cost per test	%
Capital cost	102.42	26.99
Reagents and consumables *	209.18	55.13
Personnel	46.81	12.34
Utilities	14.69	3.87
Maintenance cost of equipment	2.37	0.62
Quality assurance program	4	1.05
**Total Cost**	**379.46**	**100**

* Reagents and consumables are the most expensive inputs to HIV drug resistance testing in Viroseq HIV genotyping.

### Challenges and lessons learnt.

As a startup laboratory, the challenges and lessons learnt during the processes of establishing such a capital-intensive undertaking in a resource-limited setting were documented. Table 5 shows some of the challenges and lessons learnt.

**Table 5.** Challenges and lessons learnt

Challenges: Challenges experienced in the initial implementation of HIV drug resistance testing	Lessons learnt: Some of the solutions applied to overcome initial challenges
High staff turnover.	Building capacity through training grant application.
Insufficiencies in the supply chain management.	Strategic memorandum of understanding with the suppliers.
Lack of functional laboratory network for sample flow, hence sub-optimal utilization of the facility	Instruments suboptimal utilized can be leveraged to be used for other services.
Financial sustainability due to decreased funding.	Engagement of key stakeholders.
Frequent electricity disconnection	Need to build relationship with other laboratories.
Premixed PCR reagents kit, limiting flexibility for use with other tests	

### Sensitivity analysis

**Figure 4.**
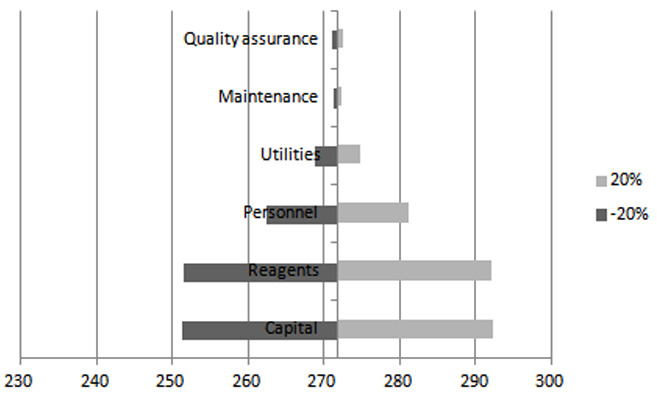
Tornado graph for one-way sensitivity analysis, costs in USD. Grey represents 20% reduction in the input costs, while dark grey represents 20% increase in the input costs. Unit cost is $271.78.

The costs presented assume that the laboratory runs 1000 HIVDR tests per year with no machine breakdown or waste of supplies. Considering that variation in input costs would have an impact on the input costs, a one-way sensitivity analysis for 20% variations to cost categories was performed. Variations to capital, reagents, and personnel inputs had a major impact on the unit cost, whereas variations to utilities, maintenance and quality assurance results had no significant impact on the unit cost. A 20% variation to capital and reagents results in changes of up to 7.5% in unit cost; approximately a $20 difference ( Figure 4).

## Discussion

The aim of this study was to establish a detailed cost profile for HIVDR testing from a provider’s perspective and identify cost drivers. We also report the challenges encountered and lessons learnt during the implementation of HIVDR testing at the MIDRL. The cost of performing HIVDR testing was estimated to be $271.78 per test. The cost estimate represents all the inputs required for performing HIVDR testing including; capital, personnel, reagents, consumables, quality assurance program and service contracts for the laboratory equipment for performing 1000 tests per year. Previous studies did not include all cost categories, making it difficult to compare costs for offering drug resistant tests across many laboratories ( Acharya *et al.*, 2014; Alemán *et al.*, 2015; Inzaule *et al.*, 2013; Novitsky *et al.*, 2015). In this study, we offer a framework for performing laboratory cost analyses that makes it easy to compare cost categories between different laboratories. A previous study from KEMRI/CDC, Kenya, performed a cost analysis of their in-house assay and established the unit cost to be approximately $113.33, with $109.31 as the cost of reagents and consumables ( Inzaule *et al.*, 2013). The analysis included the costs of reagents and consumables and the cost of maintaining the equipment but did not account for capital, personnel and external quality assurance program costs. Considering the reagent and consumable costs, there was a correlation in the cost results for these items, as our study estimated the cost to be $101.50. The slight difference could be attributed to time difference in performing the cost analysis. In addition, the previous study estimated the equipment maintenance cost to be $4.02, while the present study estimates a cost of $2.37 for this category. Some of the studies performed in other parts of the world found considerably lower reagents and consumables costs than those estimated by this study. For instance, the costs reported in India and Cuba were $85.00 and $87.80, respectively ( Acharya *et al.*, 2014; Alemán *et al.*, 2015). Conversely, one study reported higher reagent costs than those found in the present study; the estimated cost was $139.75 per test ( Novitsky *et al.*, 2015).

To answer the question of the cost drivers for HIVDR testing, the costs were categorized according to the processes involved in HIVDR testing, including sample collection, RNA extraction and amplification, gel electrophoresis, sequencing, and sequencing analysis. In terms of cost categories, capital cost took the biggest share of pie at $102.42 (37.68%), followed by reagents plus consumables at $101.50 (37.35%). High capital costs could be attributed to sub-optimal utilization of the sequencing platform. It should be noted that the equipment required for HIVDR testing can be leveraged to perform more patient tests that support HIV care and treatment, therefore bringing down the cost of equipment attributed to HIVDR testing. Our cost analysis was based on an estimated projection of 1000 HIVDR tests per year, which is a gross underestimation of the laboratory’s capacity. If the laboratory operated optimally, offering approximately ~6720 tests per year, the capital cost would reduce ~6.7 fold. The reagent costs were considerably high as a result of acquiring the sequencing machine at no upfront cost. This bound the laboratory to only procure reagents and consumables from the machine provider. This commitment denies the laboratory an opportunity to practice strategic purchasing, which would be a key factor in lowering the cost of reagents. This is not unique to HIVDR testing; a study done in Kenya to estimate cost of HIV viral load and EID reported high reagent costs as a result of machine acquisition on a placement basis ( Cintron *et al.*, 2017).

A comparison with other studies was impossible as most of the previous cost analysis included reagents and consumables costs only, omitting other categories such as capital, personnel, utilities and quality control program costs ( Alemán *et al.*, 2015; Inzaule *et al.*, 2013; Novitsky *et al.*, 2015). In terms of cost per process, the sequencing step, which involves purification of PCR products, cycle sequencing, purification of sequencing products and sequence detection, was the most costly step in HIVDR testing at $160.94 (59.22%). This is in keeping with other studies evaluating the cost of HIVDR testing. Other studies report $59.88 (52.92%) and $50 (58.82%) as the cost for the sequencing step ( Acharya *et al.*, 2014; Inzaule *et al.*, 2013). The one-way sensitivity analysis performed illustrates a cost saving opportunity, for example through negotiating lower reagent prices and maximizing utilization of the sequencing platform, therefore ensuring sustainable use of health financing resources.

Comparing the cost of the HIVDR test to a HIV viral load test used in monitoring and management of people living with HIV, the HIVDR testing cost is higher. A study in Kenya estimates HIV viral load test at $24.63 for non-point-of-care viral load testing and $29.74 for point-of-care HIV viral load testing ( Cintron *et al.*, 2017). This is attributed to additional processes in HIVDR test, that is, nested PCR and cycle sequencing processes. These increase the amount of cost inputs used in HIVDR testing, especially staff hands-on time.

The study evaluated the effects of reducing the reagents volume on the cost and performance characteristics of the HIVDR testing in view of reagents being one of the cost drivers for HIVDR testing. On the cost of the HIVDR testing, there was a significant reduction in the cost from $271.78 to $247.30; a ~13.97% reduction in the cost per test. This assay modification led to a ~37.68% reduction in reagent costs. Of note is the concordance of the two assays in their performance characteristics, which increases the confidence in adoption of this cost saving undertaking by the laboratories that would like to increase their efficiency in offering the HIVDR testing service ( Magomere *et al.*, 2019). The new assay performance characteristics met the WHO HIVDR validation criteria ( WHO, 2018). Cost computation using Viroseq reagents, which are FDA approved and an alternative to in-house Thermofisher (Illinois, US) reagents, gave a cost of $379.46 per test, with reagents taking the biggest share of the cost at 55.13% ($209.18). This illustrates a lower cost of HIVDR testing using Thermofisher reagents by $107.68. These findings are in keeping with other studies where the cost of HIVDR per test was lower when using the in-house system compared to the Viroseq system ( Acharya *et al.*, 2014; Inzaule *et al.*, 2013; Zhou *et al.*, 2011). For instance, one study reported a $132.86 difference in the two systems, while another reported a $165.01 difference ( Inzaule *et al.*, 2013).

One of the challenges encountered during the implementation of HIVDR testing was high staff turnover. This is attributed to advanced molecular skills required for sample analysis in HIVDR testing. There are a few laboratory specialists equipped with these skills, making them highly sought after in the job market. This is a challenge in a low resource set up as training personnel on this area is quite expensive ( Abimiku, 2009; Kennedy *et al.*, 2016; Nkengasong *et al.*, 2018; WHO, 2010). To counteract this challenge, one of the staff won a training grant to learn HIVDR testing from a laboratory that was already established. The sequencing machine provider is also bound by the contract to train the laboratory staff to the highest level possible and provide machine service when due. Maintaining a good working relationship with other laboratories performing the test helps in the exchange of new ideas and also facilitates an inter-laboratory proficiency testing program.

Unlike HIV viral load and EID, HIVDR testing is not included in the Global Access Program, which has helped in the scaling up of HIV viral load and EID testing in Kenya at a relatively low cost ( WHO, 2014). This raises sustainability concerns owing to recent reduced donor funding for HIV programs. However, HIVDR testing services can leverage on already established sample referral networks, human resources, laboratory equipment and database for HIV viral load. The multiple possible applications of the sequencing platform provides opportunities to deploy it for other tests and services, therefore reducing the overall running costs. Sensitization of key stakeholders involved in management of people living with HIV through regular stakeholders meetings has been instrumental in uptake of HIVDR test.

Other challenges experienced during the implementation of drug resistance testing included frequent electricity disconnections, which was solved by installing a backup generator to ensure a constant supply of power. This corroborates other previous studies that highlighted similar findings in resource limited settings ( Kennedy *et al.*, 2016; Nkengasong *et al.*, 2018). Furthermore, supply chain insufficiency, which delayed timely delivery of reagents, consumables, and laboratory equipment, was a major setback in implementing HIVDR testing. Finally, the premixed PCR master-mixes limited the flexibility of their use for other tests.

## Strengths of the study

This report presents findings from a complete cost analysis performed in the early stages of implementation of HIVDR testing, hence giving a good picture of the costs involved in the process. This report will further serve as a useful resource for planning and budgeting information for better resource management for similar projects in future. The inclusion of the cost-saving assay evaluation makes the study one of a kind, as it provides an evidence of cost reduction and comparable performance characteristics for both assays.

## Study limitations

The study estimated costs from the provider’s perspective, thus limiting the inclusion of cost incurred by patients. The study design also excluded transport costs incurred for the transport of samples from peripheral health facilities to the testing laboratory in Nairobi. Furthermore, the cost analysis was carried out in only one facility, hence hindering the comparison across facilities offering HIVDR testing. This study presents a partial economic evaluation; a complete economic evaluation would give a clearer picture on the cost-effectiveness of HIVDR testing versus the status quo. Finally, at the time of the study the laboratory was not operating at full capacity, which increases the unit cost of HIVDR testing. It is conceivable that once uptake for the HIVDR test increases, the additional volumes would translate to reduced costs.

## Conclusion

The MIDRL has implemented HIVDR testing capacity for patients failing ART at a cost of $271.78 per test. The most important cost driver is expenditure on capital cost, which is likely to reduce when utilization of the equipment increases. It has also been demonstrated that there are opportunities for cost saving through assay modifications such as selective reagent volume reduction.

## Data availability

### Underlying data

Figshare: Cost analysis of implementing HIV drug resistance testing in Kenya: a case study of a service delivery site at a tertiary level hospital in Kenya: https://doi.org/10.6084/m9.figshare.12561980.v3 ( Gachogo *et al.*, 2020a).

This project contains the following underlying data:

- HIVDR_Consumables cost.xlsx
- HIVDR_BuildingCost.xlsx
- HIVDR_Electricitycost.xlsx
- HIVDR_Equipment_cost.xlsx
- HIVDR_Indirectcost.xlsx
- HIVDR_Personnel_cost.xlsx
- HIVDR_Reagents_cost.xlsx
- HIDVR_Viroseq_consumbles.xlsx
- HIDVR_viroseq_reagents.xlsx
- HIVDR_Field_notes.pdf

### Extended data

Figshare: Cost analysis of implementing HIV drug resistance testing in Kenya: a case study of a service delivery site at a tertiary level hospital in Kenya. https://doi.org/10.6084/m9.figshare.12628031.v1 ( Gachogo *et al.*, 2020b).

- HIVDR_Questionnaire.pdf
- Interview Guide.pdf

Data are available under the terms of the Creative Commons zero “No rights reserved” data waiver (CC0 1.0 Public domain dedication).
