# Supplementary material for: Cost analysis of implementing HIV drug resistance testing in Kenya: a case study of a service delivery site at a tertiary level hospital in Kenya
Source: F1000Res. 2020 Jul 29;9:793. [Version 1] doi: 10.12688/f1000research.23379.1 (PMC7495211; doi:10.12688/f1000research.23379.1)
Supplement: Peer review history

# Peer review: Reviewer response for version 1

**DOI:** 10.5256/f1000research.25806.r71361

**Published:** 2020-09-16

**Authors:** Joses Muthuri Kirigia

**Affiliations:** 1 African Sustainable Development Research Consortium (ASDRC), Nairobi, Kenya

**Recommendation to the Editor:**

 This is an important and pertinent manuscript in the ongoing fight against HIV/AIDS. The estimated unit cost is important for planning and budgeting purposes. Therefore, I recommend acceptance after some minor suggested revisions.

**Research Article**

- Is the work clearly and accurately presented and does it cite the current literature?
  Yes.
- Is the study design appropriate and does the work have academic merit?
  Yes. It is a cost analysis of HIV drug resistance tests study in Kenya.
- Are sufficient details of methods and analysis provided to allow replication by others?
  Yes. However, I suggest that the authors explain more about the method used to estimate the annual cost of capital inputs. Please see the detailed suggestion to the authors below.
- If applicable, is the statistical analysis and its interpretation appropriate?
  The statistical analysis is not applicable for this kind of study.
- Are all the source data underlying the results available to ensure full reproducibility?
  Yes, the study references the repository (figshare) where data has been deposited.
- Are the conclusions drawn adequately supported by the results?
  Yes, the conclusions are adequately supported by the results.

Other discretionary suggestions for the authors

**Abstract**

- Under Results section (page 1), amend the last sentence slightly to read as:
  “A user-initiated modification of halving reagent volumes for some laboratory processes (amplification and sequencing) reduced the unit cost for a HIVDR test to $233.81, i.e. a 13.97% reduction.”

**MAIN TEXT**

**BACKGROUND**

- No amendment suggested.

**METHODS**

**Data collection and analysis**

- On page 3, first paragraph, the third sentence reads “Cost data were collected on annualized depreciation for capital items including laboratory equipment and furniture, long term training and information technology equipment at a depreciation rate of 10%, …”.
- ***Suggestions (A):*** I suggest that the authors explain more about the method used to estimate the annual cost of capital inputs. As Drummond *et al.* explain, capital inputs are assets that are used over periods of more than one year; and depreciate (wear out) with time. Capital costs have two components. (1) Opportunity cost: lost opportunity to invest money used to purchase a capital input to yield positive benefit, e.g. the money could have been put in a fixed deposit to earn interest income. (2). Capital input depreciates over time – thus, its value decreases the longer the life span. Thus, costing of capital inputs should capture both components.
  As Drummond *et al* explain, there are two best methods costing capital inputs: (1) To calculate the equivalent annual cost, i.e. annuitize the initial cost of purchase of equipment (or building) over its useful life (years). In calculating the equivalent annual cost, one should take into account both the capital input useful life (in years) and a discount rate.
  (2) Where competitive market exists to use the rent (per square metre) for building space, and lease price for equipment. The rental and lease price would capture both the depreciation and the opportunity cost.
  The accounting approach of dividing the price of a capital input by the length of its useful life does not capture fully the two cost components mentioned above.
  Reference: Drummond MF, Sculpher MJ, Torrance GW, O’Brien BJ, Stoddard GL. Methods for the economic evaluation of health care programmes (3 ^rd^ Edition). Oxford: Oxford University Press; 2005. ^1^
  In Drummond *et al.*, refer to pages 64 and 65 for explanation and pages 72-75 for the formula for estimating the equivalent annual cost of a capital item.
- ***Suggestion (B):*** Authors should justify the choice discount rate of 10%. The authors cite a published article that has used the same discount rate, and that would suffice.
- ***Suggestion (C):*** On page 3, first paragraph, the fourth sentence reads “Cost details were obtained from quotations, invoices and delivery notes.” Please replace the word “Cost” at the beginning of the sentence to “Price”.

**Data analysis**

- Page 4: Please consider modifying the first sentence to read as follows:
  “We developed a costing model in Excel Software (Microsoft, New York) to aid in the estimation of the unit cost and cost for the various categories and laboratory processes.”

**Sensitivity analysis**

- Page 4: The first sentence in this subsection reads “One-way sensitivity analysis for 20% variations to cost categories was performed…”. Authors could cite published studies to justify the choice of 20% variation.

**RESULTS**

- ***Suggestion (A):*** Page 4: I suggest that the authors consider moving subsection titled “HIVDR testing process” and the first sentence of subsection titled “HIVDR unit cost” to the methods section.
- ***Suggestion (B):*** Slightly amend the Title of Table 1 to read as: “ **Table 1. Cost breakdown for each category (US$)**” Please reflect the minor amendment in Tables 2, 3, and 4.
- **Suggestion (C):** The Figures 1, 2, 3, and 4 portray results that are already contained in Tables 1, 2, 3, and 4. Therefore, I suggest for authors to choose between presenting results either Tables or Figures.

**Cost of the modified HIVDR assay**

- **Suggestion (A):** Page 5: Please move the first sentence to methods section.
- **Suggestion (B): **Page 5: Insert the percentage change of “13.97%” into the second sentence. Such that it reads as follows: “The unit cost as a result of halving reagents volumes at the amplification and sequencing step was $233.81, a **13.97%** reduction from $271.78 of the original assay.”

**Challenges and lessons learnt**

- In Table 5, rephrase the fifth challenge to read as “Frequent electric power outage”. Rephrase the second lesson learnt to read as “Strategic memorandum of understanding between the laboratories and the suppliers”.  Rephrase the third lesson learnt to read as “Underutilised equipment can be used for other programmes”.
  .

**Sensitivity analysis**

- **Suggestion:** Page 5: In the sensitivity analysis subsection, please delete the first two sentences because they were already stated in Methods.

**DISCUSSION**

- **Page 5:** The ninth sentence reads as “Considering the reagent and consumable costs, there was a correlation in the cost results for these items, as our study estimated the cost to be $101.50.”
- ***Suggestion (A):***Please reword the sentence to remove the word “correlation”. I guess you want to say that your finding is very similar to those from cited studies. Correlation is a statistical term.
- ***Suggestion (B):*** **Page 6:** Please move the first sentence of the second paragraph to the Methods section. The sentence I am referring to starts as “To answer the question of the cost drivers for HIVDR testing..”.
  Page 9: In the second paragraph, the second and third sentences read as “On the cost of the HIVDR testing, there was a significant reduction in the cost from $271.78 to $247.30; a ~13.97% reduction in the cost per test. This assay modification led to a ~37.68% reduction in reagent costs.”.
- ***Suggestion (C):*** Page 9: In the Results section, I could not find the result of $247.30. The percentage change from $271.78 to $247.30 is not ~13.97% but 9.1%. Also, I wonder whether the use of the symbol “~” is appropriate. Please check.
- ***Suggestion (D):*** Page 9: Should the third sentence read as follows “Unlike HIV viral load and EID, HIVDR testing is not included in the Global Access Program, which **could have** helped in the scaling up of HIV viral load and EID testing in Kenya at a relatively low cost (WHO, 2014).” Incase, the sentence is correct as it was, please ignore this suggestion.

**CONCLUSION**

- **Page 9:** Please consider modifying the first and second sentences as follows: “The MIDRL has implemented HIVDR testing capacity for patients **with resistance to** ART at a cost of $271.78 per test. The most important cost driver is expenditure on capital **inputs**, which is likely to reduce when utilization of the equipment increases.”

Is the work clearly and accurately presented and does it cite the current literature?

Yes

If applicable, is the statistical analysis and its interpretation appropriate?

Not applicable

Are all the source data underlying the results available to ensure full reproducibility?

Yes

Is the study design appropriate and is the work technically sound?

Yes

Are the conclusions drawn adequately supported by the results?

Yes

Are sufficient details of methods and analysis provided to allow replication by others?

Partly

Reviewer Expertise:

Economic evaluation; cost-effectiveness analysis; cost-utility analysis; cost-benefit analysis; hospital and programme costing; health financing; health economics; health systems performance assessment; national health research systems

I confirm that I have read this submission and believe that I have an appropriate level of expertise to confirm that it is of an acceptable scientific standard.

## References

[1] : Methods for the economic evaluation of health care programmes, 3rd ed. *Oxford University Press*.2005;

# Peer review: Reviewer response for version 1

**DOI:** 10.5256/f1000research.25806.r71023

**Published:** 2020-09-14

**Authors:** Graeme Jacobs

**Affiliations:** 1 Division of Medical Virology, Department of Pathology, Faculty of Medicine and Health Sciences, Stellenbosch University, Cape Town, South Africa

- The study analysed the costs involved for HIVDR testing in Nairobi, Kenya. Such studies are vital to maximize the use of available resources for laboratory assays, and to help optimize the success of HIV antiretroviral therapy outcomes.
- AIDs – S should be capital letter - AIDS.
- It should be noted that adherence is also a threat to the success of ART, along with the development of resistance.
- CD4 cell counts are not recommended anymore – test-and-treat campaigns.
- Say that DTG is an Integrase Inhibitor.
- The study gives an overall adequate outline of the major costs involved for HIVDR. As newer technologies, such as Next-generation sequencing, become more freely available (and cheaper), cost analyses studies can be re-evaluated.

Is the work clearly and accurately presented and does it cite the current literature?

Yes

If applicable, is the statistical analysis and its interpretation appropriate?

Yes

Are all the source data underlying the results available to ensure full reproducibility?

Yes

Is the study design appropriate and is the work technically sound?

Yes

Are the conclusions drawn adequately supported by the results?

Yes

Are sufficient details of methods and analysis provided to allow replication by others?

Yes

Reviewer Expertise:

Virology; HIV; Infectious Diseases

I confirm that I have read this submission and believe that I have an appropriate level of expertise to confirm that it is of an acceptable scientific standard.
